# Microwave-Assisted Extraction of Polyphenols from Blackcurrant By-Products and Possible Uses of the Extracts in Active Packaging

**DOI:** 10.3390/foods11182727

**Published:** 2022-09-06

**Authors:** Federica Alchera, Marco Ginepro, Giovanna Giacalone

**Affiliations:** 1 Department of Agricultural, Food and Forest Sciences, University of Turin, Largo Paolo Braccini 2, 10095 Grugliasco, Italy; 2 Department of Chemistry, University of Turin, Via Pietro Giuria, 5, 10125 Torino, Italy

**Keywords:** circular economy, waste recover, green extraction technique, microwave-assisted extraction (MAE), sustainable packaging, raspberries storage

## Abstract

The design of experiment (DoE) approach was used to optimize the extraction of polyphenols from blackcurrant by-products with microwave-assisted extraction and deionized water as a green solvent. Three factors (microwave power, extraction time, and solvent/matrix ratio) were evauated, and a central composite orthogonal design (CCO) was applied in order to reduce experimental runs. Empirical models relating the response and process parameters were developed. The validity of the models was tested using an analysis of variance (ANOVA). The optimal extraction conditions were found using the highest value of microwave power (780–800 W) and the lowest extraction time (60 min) and solvent/matrix ratio (10 m/g). Compared with conventional solvent extraction, the polyphenol yield increased by 25% after applying the optimized MAE process. The obtained extract was used to realize a sustainable active maltodextrin (Glucidex 2) pad using an electrospinning technique. The antimicrobial and antioxidant activities of the pads were tested on the post-harvest storage of raspberries. Two set of experiments were carried out. The recorded results showed that the pad had antimicrobial activity on the tested fruit samples and implied the possibility of using it to extend the shelf-life of the fruits.

## 1. Introduction

The processing of agricultural products inevitably leads to large quantities of waste, which is a serious disposal problem. Most of the waste is recycled as animal feed and compost, but large quantities remain [1]. In particular, the fruit and vegetable supply chain results in approximately 90 million tons of residues per year in Europe, a number that is expected to grow in the coming years [2]. For instance, a large part of the total berry production is processed for juice production, and about 20–30% of that portion ends up as waste [3].

In a circular economy approach, also considering the high value due to the presence of active compounds, it is crucial to recover produced waste. In facts, fruit residues are rich in phenols and active substances. In particular, polyphenols are molecules with one or more phenol groups that are classified into different families: phenolic acids, flavonoids, stilbenes and lignans [4,5]. These molecules are known for their antioxidant [6,7] and antimicrobial properties [8,9].

The extraction of polyphenols from agri-food wastes can be carried out using different techniques, including conventional liquid extraction (e.g., mechanical agitation, maceration, and Soxhlet) and more efficient methods (e.g., ultrasound-assisted extraction (UAE), microwave-assisted extraction (MAE), pressurized liquid extraction (PLE), and supercritical fluid extraction (SFE)) [10,11]. The development of a polyphenol valorization process requires the identification of the most efficient extraction method, operating conditions, and compatible solvents [12,13,14].

From the perspective of a circular economy, polyphenol extracts can be used as active agents, for instance, in active packaging. This type of packaging makes it possible to extend shelf life by maintaining the condition of packaged foods through interactions with the food itself. Generally, packaging consists of a synthetic polymer into which active agents are incorporated. However, researchers are evaluating more environmentally sustainable solutions for packaging production by exploiting the use of biopolymers. Prominent among biopolymers are the maltodextrins, water-soluble carbohydrates that are obtained by chemical hydrolysis processes, mainly from the breakdown of cereal or tuber starches. Those products are classified as biopolymers that are both bio-based and biodegradable [15]. These polysaccharides have attracted increasing scientific and industrial interest, and they have been applied in industrial processes such as packaging and bio-composite production [16,17,18]. In particular, starch derivatives, such as maltodextrins, have recently proven to be powerful tools for the encapsulation and slow release of flavors and food preservatives [19]. Maltodextrin pads have been created with an electrospinning technique that has evident advantages, mainly related to the high surface-to-volume ratio of the produced mats [20]. However, in many cases, a limitation is represented by the need for toxic or flammable organic solvents, which can limit industrial production due to environmental and safety regulations. In this regard, developing processes aimed to reduce or eliminate the use of hazardous substances represents one of the key points of green chemistry. For this reason, several researchers are working on the possibility of obtaining fibrous mats by electrospinning water-soluble maltodextrin to avoid the use of any organic solvent. The high solubility of these bio-based precursors in aqueous environments makes it possible to create a green process, but a cross-linker such as citric acid must be added to obtain an insoluble fiber [21].

The aim of this study was to evaluate the possibility of performing the green extraction of polyphenols from blackcurrant residues from the fruit juice industry, using only deionized water as solvent. Microwave-assisted extraction (MAE) was used to improve extraction efficiency and capability. The goal was to obtain an extract from the polyphenol-rich processing waste that could be used to realize a sustainable active pad comprising maltodextrins. The antimicrobial and antioxidant properties of this pad, as well as its potential to extend storage, were tested on raspberries.

## 2. Materials and Methods

### 2.1. Extraction Process

#### 2.1.1. Raw Materials

Blackcurrant by-products, coming from a juice production company located near Torino, Italy, were used as extraction matrices. Samples were stored at −20 °C until use.

#### 2.1.2. Optimization of Microwave-Assisted Extraction (MAE) of Polyphenols from Blackcurrant Residuals (Experimental Design)

The extraction process was performed using only deionized water as the solvent (ε = 80 and tanδ = 0.123 at 20 °C) [22]. Samples were treated using a microwave extractor (Ethos UP, Milestone Connect, Bergamo, Italy). The microwave extractor was equipped with two magnetrons for a total of 1900 Watts, and the magnetron frequency used was 2.45 GHz. Various experimental conditions were tested:

- Extraction time (from 60 to 180 min).
- Power of microwave (from 500 to 700 W).
- Solvent/solid sample ratio (from 10 to 15 mL/g).

In each experimental assay, 50 g of raw material was mixed with the correct ratio of the solvent and was placed in an extraction vessel. After MAE treatment, the resulting extract was separated from the solid part with a sieve. The supernatant was centrifuged (centrifuge Hermle Z 380, Ghosheim, Germany) for 10 min at 3500 rpm and stored at 4 °C until analysis (not longer than two days). Extractions were performed in triplicate; extracts were kept at −20 °C.

The design of experiments software (Modde Go 13, Sartorius Umetrics, Gottingen, Germany) was used to optimize the MAE. After the factors and responses of interest were defined, an appropriate design type was selected and an experimental worksheet table was generated by applying a central composite orthogonal design. Then, a response surface model (RSM) was used [23].

The experimental design employed in this work had three numerical factors: the power of the microwave, the extraction time, and the solvent/matrix ratio. As optimized response, the total phenol content (TPC) in the extract was measured. The phenol concentration was expressed as g of gallic acid equivalent (GAE)/L. The focus was on the phenol concentration in the extract because the objective was a ready-to-use product with the highest concentration of the active compound. In this study, the dependent variable was the TPC, and the independent variables were extraction time (A), power of the microwave (B), and solvent/matrix ratio (C).

The applied CCO (central composite orthogonal) design produced 17 experimental runs. The experiments were carried out in a randomized order. The CCO experimental design was chosen due to its ability to estimate second-order response surfaces and resolve quadratic terms in the response surface model.

Table 1 shows the run order, experimental design, and the observed response for the three variables and 17 experimental runs generated.

The optimization procedure was performed after model refinement with the aim to define the processing conditions for the highest concentration of polyphenols in the extract; optimizer function of Modde software was used.

For each sample, the antioxidant capacity (FRAP method) was evaluated. In order to test the solvent extraction capability, the TPC from the MAE extract was compared with the TPC value of the organic solvent extraction.

#### 2.1.3. Determination of TPC and Antioxidant Activity of the Extract

The determination of the TPC was performed using the Folin–Ciocalteu [24] method, and the antioxidant activity was determined with the FRAP method [25]. The spectrophotometric measurements (V-550 spectrophotometer, Jasco, Easton, MD, USA) were carried out at the selected wavelengths (765 nm for TPC and 595 for FRAP) using a double-beam system in which test and blank solutions were placed in the sample and reference holders, respectively. For the evaluation of TPC, a standard calibration curve was plotted using gallic acid at concentrations of 0.4–2 g/L, and the results are expressed as g of gallic acid equivalents (GAE)/L. FRAP is expressed as mmol Fe^2+^/kg. All the used chemicals were of analytical grade.

#### 2.1.4. Extraction with Organic Solvent

The TPC of the MAE extract was compared with the TPC of the extract obtained with the organic solvent. For the extraction, 10 g of samples was weighed in a centrifuge tube after adding 25 mL of extraction solution (500 mL of methanol, 24 mL of water, and 1.4 mL of HCl 37%). After 120 min in the dark, the extracts were manually homogenized for about 1 min and then centrifuged for 15 min at 3500 rpm (Hermle Z 380 centrifuge, Ghosheim, Germany) [26]. Results are expressed as mg GAE/g dry matrix considering the volume of solvent and grams of matrix used for both extractions (MAE and organic solvent extraction). Extractions were performed in triplicate.

### 2.2. Active Pad Production

#### 2.2.1. Active Pad Production and Activation with Phenol Extract

Pad production was performed with an electrospinning technique from maltodextrins. This technique was used to provide microfibers from a liquid solution. The fibers were realized with commercial Maltodextrin Glucidex 2 (Roquette Freres, Lestreme, France), obtained via the partial hydrolysis of starches of corn (DE equal to 2 and average molecular weight of about 314,000 Da) and crosslinking by citric acid.

The solution was loaded into a 3 mL syringe (BD, Sigma-Aldrich, Darmstadt, Germany) equipped with an 18 gauge PrecisionGlide needle (BD, Sigma-Aldrich, Darmstadt, Germany) for the electrospinning process. The needle of the syringe was connected to a high-voltage DC generator (Glassman High Voltage, Pulborough, West Sussex, UK) set to a voltage of 30 kV. The collector for the electrospun fibers was a rotating drum (cylinder-shaped, length of 120 mm, and diameter of 80 mm) covered with a paper sheet. The solution was ejected from the syringe with a controlled feeding rate, set to 20 μL/min. The working distance (from the tip of the needle to the collector) in all preparations was 15 cm, and all the electrospinning processes were conducted at room temperature.

During the electrospinning process, baking paper was used as fiber support. After the electrospinning process, a 30 min heat treatment at 180 °C was conducted to turn the fibers insoluble.

Pads were activated with the most concentrated extract obtained from the MAE optimization (with the highest value of TPC expressed as g/L).

Two types of pads were made:

- Pad F_0_: The electrospinning solution included maltodextrin and water at a 1:1 ratio (wt%) and citric acid as a cross-linker (16% wt of the amount of Glucidex). Active pads were created by impregnation with the phenol extract of the insoluble microfibers obtained as explained before. For the impregnation process, 3 mL of polyphenol extract was used.

The obtained fibers were previously saturated with deionized water to simplify the obtainment of the layer. After this treatment, fibers were placed in a ventilated oven at 60 °C for about 45–50 min.

- Pad F_1_: The phenol extract was added to the electrospinning solution that included maltodextrin, water, and extract at weight ratios of 1:0.6:0.3 (wt%), respectively, as well as citric acid (16 wt% of the amount of Glucidex).

To avoid the degradation of polyphenols in the fiber, the heat treatment was carried out in a tubular oven (Carbolite TZF 12/62/550, Carbolite Co. Ltd., Derbyshire, UK) in nitrogen flow for 30 min at 180 °C. After the heat treatment, the pad was already activated.

Obtained pads were cut to 1 dm^2^ squares and used as active pads.

#### 2.2.2. Morphological Characterization (Scanning Electron Microscope, SEM)

The morphology characterization of the fibers was performed using a scanning electron microscope (SEM) (Vega, Tescan, Brno, Czech Republic). Samples were prepared by taking a portion of the microfiber pads and placing it on a stub (specimen holder) to which a small strip of conductive carbon tape was previously applied. A secondary electron detector was used for the analysis by setting a beam acceleration voltage of 3 KeV. The morphology of the fibers was evaluated from the obtained images, and the identification of any morphological defects and dimensional analysis were performed using ImageJ software.

### 2.3. Application Test of Active Pads on Fruit Packaging

#### 2.3.1. Fruit

Raspberries (cvs Clarita and Grandeur) were provided by Ortofruit Italia soc.agr. Coop OP (Lagnasco, CN, Italy). The fruits were hand-picked from commercial orchards located in Cuneo Province. Each fruit was selected for its uniformity of ripeness and size, as well as the absence of physical injuries and microbial infections.

#### 2.3.2. Application Test

The raspberries were randomly packed in PET clamshells, with approximately 150 g of fruit in each (1 per treatment/each analysis date, with 19 clamshells in total). Two sets of experiments were carried out.

The compared treatments for the first set were as follows:

- Control: Clamshell containing raspberries cv Clarita without the active pad (CP).
- F_0_: Clamshell containing raspberries cv Clarita with active pad F_0_.

The samples were kept in dark cold storage (4 °C) for 13 days. Analyses were performed at the beginning of the trial (day 0) and then after 3, 6, 9, and 13 days. The evaluations were performed at the same temperature: room temperature of 20–25 °C.

The compared treatments for the second set were as follows:

- Control: Clamshell containing raspberries cv Grandeur without the active pad (CP).
- F_0_: Clamshell containing raspberries cv Grandeur with active pad F_0_.
- F_1_: Clamshell containing raspberries cv Grandeur with active pad F_1_.

The samples were kept in the dark at 15 °C for 5 days and then at room temperature for 3 days. Analyses were performed at the beginning of the trial (day 0) and after 3, 5 and 5 + 3 days.

The evaluations were performed at the same temperature: room temperature of 20–25 °C.

#### 2.3.3. Fruit Analysis

Quality, visual, and nutraceutical analyses were performed to evaluate fruit quality evolution during storage and among different treatments.

To test the possible antimicrobial effect of the pads, the incidence of diseased fruit on each date of analysis and for each treatment was evaluated. Diseased fruits are expressed as the % weight of total weight.

The visual evaluation of raspberries was performed on each analysis date. The evaluations (based on color, shriveling and freshness) were conducted using a 1–5 visual rating scale [27].

Weight loss was determined by weighing the fruit at day 0 and then at each analysis date. Results are expressed as the percentage of weight lost relative to the initial weight, according to Equation (1):

WL (%) = 100 × (W0 – Wt)/W0(1)

where WL is the percentage of weight lost, W0 is the initial weight of the fresh fruit sample, and Wt is the weight of the fruit sample at time t.

The total soluble solid content (TSS) was determined on clear raspberry juice using a digital refractometer (ATAGO-PR-32, Milano, Italy). The analysis was conducted in triplicate, and the results are expressed in ° Brix.

Nutraceutical analyses of the total phenol content (TPC) were performed on each date of analysis. The clear juice of raspberries samples was extracted by adding 12.5 mL of MeOH (100%) to 5 g of fruit and homogenizing at 24,000 rpm for 1 min using an Ultra-Turrax T18 basic (Janke and Kunkel, IKA^®^-Labortechnik, Saufen, Germany). Total phenolic content was determined with the Folin–Ciocalteu reagent, using gallic acid as a standard. Absorption was measured at 760 nm. Results are expressed as mg gallic acid equivalents (GAE) per 100 g of fresh weight. Three replicates of each treatment were performed.

### 2.4. Statistical Analysis of the Experimental Results

The design of experiments, analysis of results, prediction of responses, and statistical analyses were carried out using Modde 13 and Minitab Statistical Software (Unimetrics, Sweden). In the application test on raspberries, an ANOVA was applied among treatments for each storage time using Statistica software (Statistica 10.0, Statsoft Inc., Tulsa, OK, USA). The sources of variation were the treatment and storage time. Means were compared using Tukey’s test (*p* ≤ 0.05).

## 3. Results and Discussion 

### 3.1. Model Fitting and Refinement

The applied design allowed for the choice of a mathematical model (quadratic or linear regression) that best described the relation and the interaction among factors.

The experimental data were fitted to a polynomial equation, as this method is suitable when there is only one response at a time, and then the model was refined via the removal of nonsignificant coefficients.

**Figure 1:**
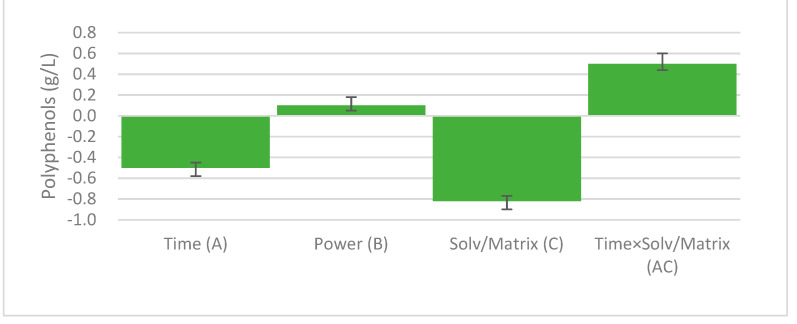
Effects of process variables in microwave-assisted extraction of polyphenols from blackcurrant waste. A: Time of extraction; B: power of microwave; C: solvent/matrix ratio; AC: power of microwave and time of extraction interaction term. N: number of experiments; *R*^2^: coefficent of determination; RSD: Residual Standard Deviation; DF: Degrees of Freedom; *Q*^2^: goodness of prediction; Confidence: confidence interval. N = 12, *R*^2^ = 0.996, RSD = 0.7465, DF = 7, *Q*^2^ = 0.983, Confidence = 0.95.

The factors under study were extraction time (A), power of the microwave (B), and solvent/matrix ratio (C). Interaction factors and quadratic factors were also included. The coefficient plot of the effects was used to select the significant factors that influenced TPC in the extract and included in the model (Table 2; Figure 1). A *p*-value < 0.05 indicates significant terms.

As shown in the Table 2, the significant coefficients were time of extraction (A), power of microwave (B), solvent/matrix ratio (C), and the interaction between solvent/matrix ratio and time of extraction (AC). So, the model could be described by a multiple linear regression with the following Equation (2):

Y = β_0_ + β_1_ × X_1_ + β_2_ × X_2_ + … + β*n* × X*n*+ε (2)

where Y is the dependent variable; β_0_, *n* = 0, 1, 2…k are the regression coefficients of the model; and X1,*n* = 1, 2, …k are the independent variables of the model.

The graph reported in Figure 1 (coefficient plot) shows the significant terms and their coefficients. The plot displays the regression coefficients with their standard deviation. All the terms reported are significant.

The coefficients of the polynomial equation that represents the model were obtained from data reported in Figure 1 (Equation (3)):

Y = 2.776 − 0.54 A + 0.129 B − 0.87 C + 0.556 AC(3)

Finally, an analysis of variance (ANOVA) was used to test the regression significance, and the model’s lack of fit was assessed with *F*-tests (Table 3).

The model was significant at a confidence level of 95%, and no lack of fit was observed (*F*-value of 7.53 and *p*-value of 0.12), i.e., the model error was smaller than the pure error. An R^2^ value of 0.997 and an R^2^ adj. value of 0.994 indicate a good correlation, and a Q^2^ value of 0.9958 indicates a goodness of fit for the polyphenol’s response.

### 3.2. Optimization of Polyphenol Extraction and Model Verification

**Figure 2:**
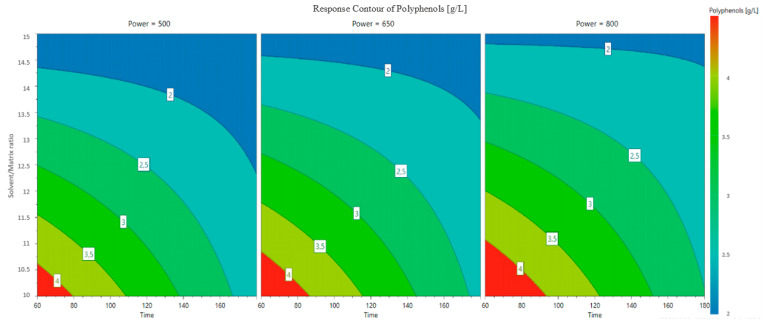
Contour plot. Power set was used to estimate the change in polyphenols as a function of time and solvent/matrix ratio. From left to right, the power was set to 500, 650 and 800 W. The red zone represents the conditions for obtaining the highest concentration of polyphenols.

**Figure 3:**
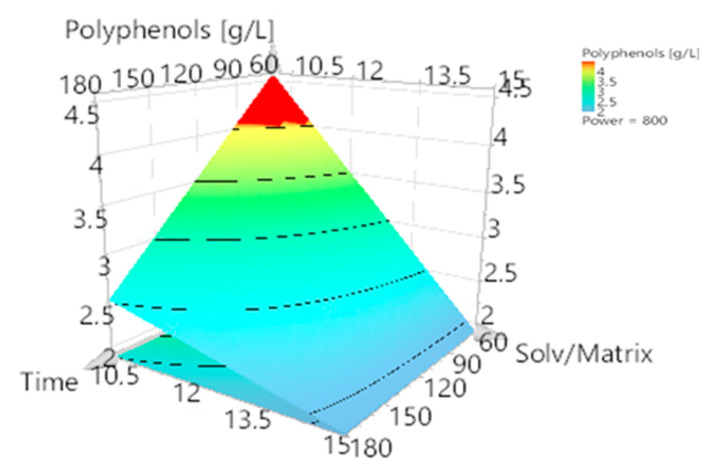
Response surface plot. Power of microwave was set to 800 W. From blue zone to red zone, the concentration of polyphenols increases.

Based on the constructed mathematical model, response surfaces and contour plots were generated to evaluate the effect of processing factors on the phenol content of the extracts. The contour plot is reported in Figure 2, where the graphs evaluate the influence of the three factors on the concentration of polyphenols in the extract. In Figure 3, a response surface plot is reported and enables the evaluation of the optimized conditions for extraction.

As shown in the contour plot (Figure 2), the concentration of polyphenols in the extract increased with the reduction in extraction solvent and the time of extraction regardless of the set power. In general, a high solvent/sample ratio cause lower heating, as the solvent mostly absorbs the microwave radiation, whereas a low ratio creates mass transfer barriers [28]. In this application, it was found that it was better to use less solvent to obtain an extract with a higher concentration of ready-to-use polyphenols. The use of less solvent involved the use of greater microwave power, but the operating times were the lowest of those tested.

After the evaluation of the contour plot, the response surface plot was built with the extraction-optimized condition of 800 W. As noted in Figure 3, the polyphenol yield decreased with higher extraction times and solvent/matrix ratios. When a lower solvent/matrix ratio was used, a lower extraction time was used to reach the highest total phenol content yield. This is in accordance with the model, which showed that the extraction time and the solvent/matrix ratio had a negative effect, though there was a positive interaction effect of these factors, as described in Figure 1 and optimized equation. Indeed, when low amounts of extraction solvent were used, it took less time to heat them and consequently resulted in extracts with higher concentrations of polyphenols in a shorter time.

Finally, analyzing both graphs (Figure 2 and Figure 3) shows that the better working conditions were in the same chart area: 60 min of extraction and a solvent/matrix ratio of 10 g/mL (the lowest amount of solvent). When working at 500 W, a solvent/matrix ratio of 10 mL/g used for 60 min led to a TPC of 4.28 ± 0.06 g/L; at a microwave power of 800 W, the obtained concentration was 4.64 ± 0.07 g/L.

Optimal working conditions were sought using the optimization function of the Modde software. The optimal working conditions were the same as those found when analyzing the previous graphs: 60 min, 780 W, and 10 mL/g reaching a predicted TPC value 4.38 g/L.

Further duplicate tests were conducted to verify the accuracy of the model by applying these parameters. The values obtained for the polyphenol content were 4.40 ± 0.21 and 4.61 ± 0.1 mg/L, which were close to the predicted value in the optimization process, so the model was adequate.

### 3.3. Antioxidant Capability: FRAP Method

For each obtained extract, antioxidant activity was evaluated with the FRAP method. Pearson correlation coefficients (r) between FRAP values and the three main factors (solvent/matrix ratio, time of extraction, and microwave power) were calculated (Table 4).

There was a negative correlation between the solvent/matrix ratio and the FRAP values: as the solvent/matrix ratio decreased, the FRAP value increased. This correlation agreed with the previous observations, in which the concentration of polyphenols in the extract increased as the solvent/matrix ratio decreased.

The FRAP values, obtained in the extract using better extraction conditions, were 61.33 ± 0.12 (mmol Fe^2+^/kg). These results are in agreement with those found in the literature [29,30].

### 3.4. Solvent Extraction Efficiency: Comparison of Microwave-Assisted Polyphenol Extraction with Conventional Method Extraction

The solvent extraction efficiency was evaluated by comparing the TPC values obtained from the optimized MAE with the TPC value from conventional organic solvent extraction. For this purpose, the TPC values obtained from the MAE extract expressed as g (GAE)/L were converted to mg GAE/g dry matrix considering the solvent/matrix ratio used for the extraction. Analyses were performed in triplicate.

In Table 5, the TPC values (mgGAE/g dry matrix) obtained with the two methods of extractions are reported.

Results demonstrated significant differences between the two methods, with higher values of TPC for the MAE (Table 5). The results are consistent with those of several authors [31,32], and the use of water as a solvent enabled the obtainment of a ready-to-use extract without needing to remove the solvent before use.

### 3.5. Characterization of Microfibers and Active Pad

#### Scanning Electron Microscopy (SEM)

**Figure 4:**
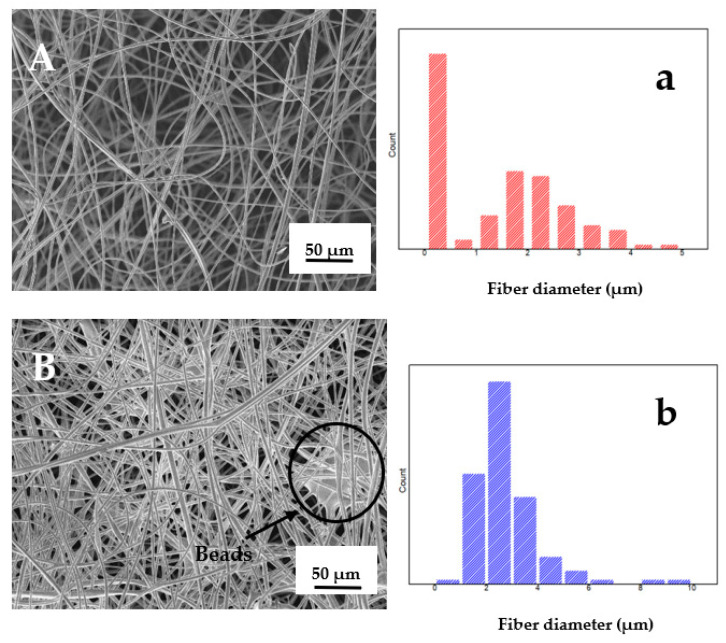
SEM micrographs of pads fibers and histograms showing the fiber diameter distribution: pad F_0_ (**A**) and (**a**); pad F_1_ (**B**) and (**b**).

The morphological feature and the dimensional analysis of the two fibers (F_0_ and F_1_) are reported in Figure 4.

As can be seen, there was a morphological difference between the two types of fibers. F_1_ showed “beads” (morphological defect circled in image B), which indicates excessive dripping and leads to a decrease in mechanical properties [33,34]. In F_0,_ on the other hand, the absence of “beads” was evident. Such morphological defects were due to difficulties encountered during electrospinning due to the viscosity of the starting solution containing the extract.

Finally, using ImageJ and Origin software, it was possible to perform the size and size distribution analyses reported in the graph of Figure 4a,b. Each image was divided into four quadrants, and for each of them, 25 different fiber diameters were randomly taken for a total of 100 values per image. The recorded data showed that the average diameter of F_0_ (1.36 µm ± 1.29) was lower than the diameter of F_1_ (2.79 µm ± 1.32) due to the abovementioned solution viscosity.

**Figure 5:**
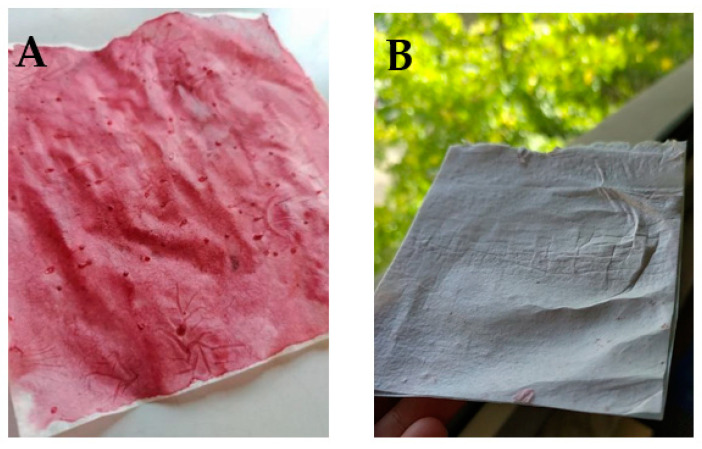
Image of created active pads: pad F_0_ (**A**) and pad F_1_ (**B**).

Figure 5 shows photos of the two obtained pads (F_1_ and F_0_).

### 3.6. Evaluation of the Effects of the Active Pad on Raspberries

**Figure 6:**
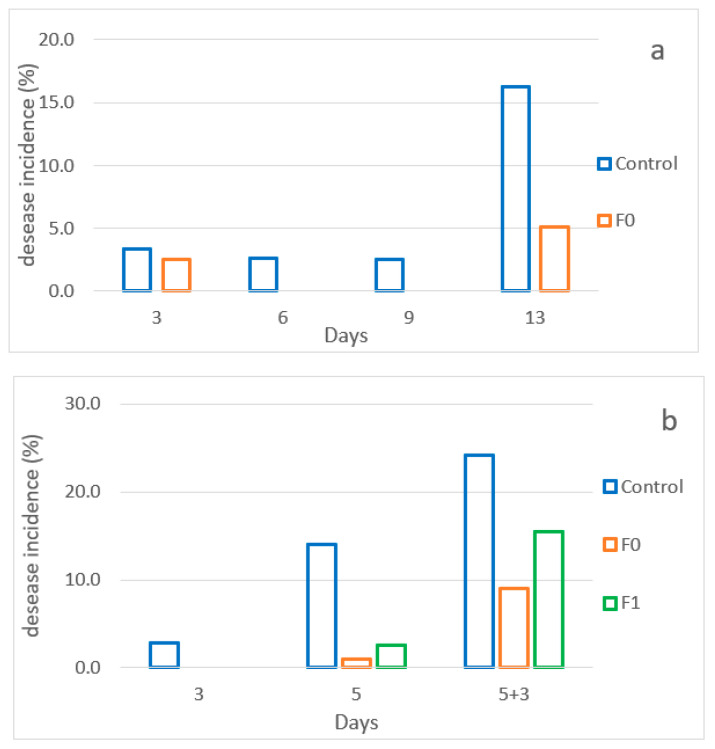
Decay incidence (%) per treatment/each analysis date. Control: Clamshell containing raspberries without the active pad (CP). F_0_: Clamshell containing raspberries with active pad F_0_. F_1_: Clamshell containing raspberries with active pad F_1_. (**a**) shows the desease incidence for the first set of analysis after 3, 6, 9, 13 days. (**b**) shows the desease incidence for the second set of analysis after 3, 5 and 5 + 3 (after 3 days at room temperature) days.

Analyzing the effect of the two different pads on raspberries showed the same differences as predicted. For each set of experiments, disease incidence (%), visual assessment, weight loss (%), total soluble content (TSS), and total phenol content (TPC) were evaluated. Only relevant results are reported. Differences among the treatments were observed for fruit disease. In the control fruits, the percentage of decay was higher than in the packaging with pads in both the first and second tests, with an increase in decay throughout storage. The F_0_ pad seemed to be more effective at containing rot (Figure 6a,b). These results suggest that the active pads presented an antimicrobial effect.

Results were also supported by the visual assessment. For both sets of trials, visual values decreased over time but raspberries stored with the active pad showed higher values compared with control fruits.

In order to analyze storage performance, it was also useful consider data related to weight loss. Results of the second set of analysis are reported in Table 6.

The packaging with the F_1_ pad showed the lowest weight loss, and the other two treatments were similar with regard to this parameter. Thus, the F_0_ pad was not sufficiently effective in containing the water loss of fruits. These results, coupled with the high sugar content (Table 7) and acid content (data not reported) in the last few days of analysis, indicate improvements in storage conditions. The sugar content decreased in all treatments during the storage, but the decrease was lower in raspberries packed with the active pad. During the cold storage, the respiration rate was controlled by the low temperature. However, when the fruits were exposed to higher temperatures (day 5 + 3), the respiratory metabolism increased, also favoring the consumption of sugars. Considering the recorded data, it was assumed that there was a lower respiration rate in the fruit.

In the second set of experiments, there was also a particular trend of polyphenol content. Data are reported in Table 8.

The trend of the polyphenol content in the analyzed raspberries was different depending on the storage conditions. In the fruits stored in the control packaging and in the packaging with F_1_, there were increases in values, with the maximum values obtained after 3 days at room temperature. Otherwise, in the packaging with the F_0_ pad, the highest values were detected on day 5.

Comparing the three treatments, significant differences emerged at each date of analysis, and the control fruits always showed the lowest TPC during cold storage. At the last analysis date, after three days at room temperature, a more pronounced increase in polyphenols was recorded in the control fruit compared with the other two treatments. Increases in the concentration of phenolic compounds in fruits have been previously associated with exposure to biotic and abiotic stresses, the ripening process, or even the senescence stage [35,36,37]. Because raspberries are non-climacteric fruits [38], changes in polyphenol content could be due to stress conditions. In this case, the increase in polyphenols was linked to the storage temperature. The higher polyphenol content in the control fruits on the last day of analyses (day 5 + 3) indicated that the fruits were more stressed or more senescent than the other when the temperature was higher, though the same conclusions could not be reached when the temperature was low. Nevertheless, active packaging in extreme conditions seems to be the best for storage [39].

## 4. Conclusions

Microwave-assisted extraction was found a suitable and effective method for extracting polyphenols from blackcurrant industrial waste. The optimum conditions for MAE were determined as follows: power of microwave of 800 W, extraction time of 60 min, and solvent/matrix ratio of 10 mL/g. These process conditions were determined using response surface methodology to generate the maximum concentration of polyphenols in the extracts as a response. The optimized extraction conditions resulted in 4.64 ± 0.07 g(GAE)/L of extracts. Compared with conventional solvent extraction, the polyphenol yield was increased by 25% after applying the optimized MAE process, and the extraction time needed to reach maximum achievable yield was reduced from 120 to 60 min. Considering the shorter process time, the lower demand for solvent, and the higher yield of extracted polyphenols, the MAE process was proven to be useful in the recovery of heat-resistant bioactive components from food industry by-products. The lower demand for solvent also benefits the further processing of the polyphenol extracts. In addition, the use of water as an extraction solvent allows the obtainment of a ready-to-use extract. Furthermore, the use of MAE enables increases in the efficiency of water extraction compared with extraction conduced in water without microwaves.

The obtained extract could be applied in the packaging industry. An application as an active pad was tested. The key points of this application are the use of a green pad (maltodextrin) and activation with a natural MAE extract. A natural pad can be generated with an electrospinning process: an innovative, effective, electrodynamic production process used to produce fibers with small diameters. The obtained fibers are insoluble, with few morphological defects and diameters in the micrometer range. This active pad can be made via the impregnation of the obtained fibers or directly through the electrospinning process.

The antimicrobial capacity of the realized pad was tested on raspberries. After two sets of experiments, antimicrobial activity was detected.

In addition, the recorded results suggest the possibility that such a pad also results in fruit shelf-life extension.

Further research should be focused on reducing extraction time (probably working under pressure). In addition, more tests should be conducted to evaluate the pad’s efficiency for other fruits.

## Figures and Tables

**Table 1:** Experimental design for the MAE of polyphenols from blackcurrant by-products. A, B, and C are the factors, and TPC is the optimized response.

Runs	A Time (Min)	B Microwave Power (W)	C Solvent/Matrix (mL/g)	Response TPC ^1^ (g/L)
N1	60	500	10	4.28 ± 0.06
N2	60	800	10	4.64 ± 0.07
N3	180	500	10	2.24 ± 0.1
N4	180	800	10	2.58 ± 0.03
N5	60	500	15	2.52 ± 0.08
N6	60	800	15	1.85 ± 0.25
N7	180	500	15	2.22 ± 0.07
N8	180	800	15	1.9 ± 0.39
N9	120	447.03	12.5	2.55 ± 0.35
N10	120	852.97	12.5	2.08 ±0.24
N11	38.81	650	12.5	1.82 ± 0.18
N12	201	650	12.5	1.89 ± 0.42
N13	120	650	9	2.03 ± 0.09
N14	120	650	15.88	1.58 ± 0.27
N15	120	650	12.5	2.55 ± 0.53
N16	120	650	12.5	2.61 ± 0.05
N17	120	650	12.5	2.56 ± 0.07

^1^ Total phenol content.

**Table 2:** *p*-value and *F*-value for model factor.

Polyphenols	*F*-Value	*p*-Value
Coefficient ^1^		
A	958.09	0.001
B	118.55	0.008
C	2913.12	0.000
A^2^	14.09	0.064
B^2^	6.38	0.127
C^2^	2.28	0.270
AB	0.10	0.785
AC	1077.12	0.001

^1^ Model factors are A: time; B: power; C: solvent/matrix ratio.

**Table 3:** Analysis of variance for the model in the extraction of polyphenols from blackcurrant waste.

Polyphenols	DF ^a^	SS ^b^	MS (Variance) ^c^	*F*	*p*	SD ^d^
Total	12	90.99	7.58			
Constant	1	81.28	81.28			
Total corrected	11	9.72	0.88			0.94
Regression	5	9.68	1.94	350.14	0.00 ^e^	1.39
Residual	6	0.03	0.01			0.07
Lake of fit	4	0.03	0.01	7.53	0.12 ^e^	0.09
Pure error	2	0.00	0.00			0.03

*R*^2^ = 0.997 *R*^2^ adj = 0.994 *Q*^2^ = 0.9658. (^a^) Degrees of freedom; (^b^) sum of squares; (^c^) mean square; (^d^) standard deviation; (^e^) *p* ≤ 0.05.

**Table 4:** Correlation analysis between FRAP values of the extract and the three factors: solvent/matrix ratio, time of extraction, and microwave power. Pearson’s correlation coefficient (r) and *p*-value of correlation (mean of three reps) are shown.

Extraction Factor	FRAP ^1^ (mmol Fe^2+^/kg) r	*p*-Value
Solv/matrix	**−0.540**	0.025
Time	−0.193	0.458
Power	0.014	0.958

^1^ Fluorescence recovery after photobleaching.

**Table 5:** TPC (mgGAE/gdry matrix) obtained with the optimized MAE and the conventional method (organic solvent) from blackcurrant by-products. Different letters indicate significant differences for *p* < 0.05 (means of three reps).

Extraction Method	TPC ^1^ (mgGAE/gdry Matrix)
Organic Solvent	31.25 ± 0.41 B
Water solvent (MAE ^2^)	41.77 ± 0.6 A

^1^ Total phenol content. ^2^ Microwave-assisted extraction.

**Table 6:** Weight loss (%) per treatments/each analysis date.

	Day 3	Day 5	Day 5 + 3
Control	2.2	4.7	10.4
F_0_	2.2	4.7	10.6
F_1_	1.9	3.8	9.2

Control: Clamshell containing raspberries without the active pad (CP). F_0_: Clamshell containing raspberries with active pad F_0_. F_1_: Clamshell containing raspberries with active pad F_1_.

**Table 7:** R.S.R. per treatments/each analysis date.

TSS ^1^ (°brix)					
	Day 0	Day 3	Day 5	Day 5 + 3	
Control	10.1 ± 0.1 B	10.5 ± 0.1 A	10.7 ± 0.1 bA	8.5 ± 0.1 cC	*
F_0_	10.1 ± 0.1 B	10.5 ± 0.3 B	11 ± 0.1 aA	9 ± 0 bC	*
F_1_	10.1 ± 0.1 B	10.8 ± 0.3 A	11 ± 0.1 aA	9.6 ± 0.1 aC	*
		n.s.	*	*	

^1^ Total soluble solid content. Control: Clamshell containing raspberries without the active pad (CP). F_0_: Clamshell containing raspberries with active pad F_0_. F_1_: Clamshell containing raspberries with active pad F_1_. Different letters indicate significant differences for *p* < 0.05. Lowercase letters in the same column indicate statistical differences among treatments. Capital letters in the same row indicate statistical differences among storage days. n.s. not significant, * *p* < 0.05 (means of three reps).

**Table 8:** TPC per treatments/each analysis date.

TPC ^1^ (gGAE/L)					
	Day 0	Day 3	Day 5	Day 5 + 3	
Control	1.39 ± 0.01 C	1.14 ± 0.0 cD	1.66 ± 0.03 cB	2.06 ± 0.01 aA	*
F_0_	1.39 ± 0.01 D	1.64 ± 0.01 aB	1.67 ± 0.01 bA	1.59 ± 0.03 cC	*
F_1_	1.39 ± 0.01 D	1.51 ± 0.05 bC	1.7 ± 0.02 aB	1.79 ± 0.01 bA	*
		*	*	*	

^1^ Total phenol content. Control: Clamshell containing raspberries without the active pad (CP). F_0_: Clamshell containing raspberries with active pad F_0_. F_1_: Clamshell containing raspberries with active pad F_1_. Different letters indicate significant differences for *p* < 0.05. Lowercase letters in the same column indicate statistical differences among treatments. Capital letters in the same row indicate statistical differences among storage days. n.s. not significant, * *p* < 0.05 (means of three reps).

## Data Availability

The data presented in this study are available on request from the corresponding author.
